# CALENDAR

**DOI:** 10.1054/bjoc.1999.1070

**Published:** 2000-02-21


					26 March - 1 April 2000
European School of Genetic Medicine Scientific
Programme of the 13th Course in Medical Genetics
Genova, Italy
Course programme:
26 March 2000 Introduction to Genetic Analysis
27 March 2000 Genetics of Complex Traits
28 March 2000 Molecular Genetics
29 March 2000 Clinical Genetics
30 March 2000 Neurogenetics
31 March 2000 Genome Projects
1 April 2000 Genetic Medicine in Year 2000
Further information from:
Alessandro Palmesino, European Genetics Foundation, Palazzo
del Melograno, Piazza Campetto 2/8, 16123 Genova, Italy.
Tel: + 39 010 246 46 46, + 39 010 246 41 66; Fax: + 39 010 246
60 55; E-mail: eurgef@tin.iti; Website: http://www.eurogene.org
5-9 April 2000
Eurogin 2000
Global Challenge of Cervical Cancer Prevention
4th International Multidisciplinary Congress
Paris, France
Further information from:
Congress Secretariat, BAXON, N. Duraincie, C Boussin,
220-224 boulevard Jean, Jaures, 92773 Boulogne Cedex, France.
Tel: + 33 (0) 15520 23 83; Fax: + 33 (0) 1552023 93;
E-mail: duraincie.baxon@covos.fr
11-13 April 2000
MICRO 2000 - International Microscopy Conference &
Exhibition
London, UK
Further information from:
MICRO 2000, Royal Microscopical Society, 37/38 St Clements,
Oxford OX4 1AJ, UK. Tel: +44 (0) 1865 248768; Fax: +44 (0)
1865 791237; E-mail: jenny@rms.org.uk;
Webpage: www.rms.org.uk/mic2000.html
27 September - 1 October 2000
Pigment Cell research - Perspectives for the Third
Millenium
University of Ulm/Donau, Germany
Further information from:
Department of Dermatology, University of Ulm, ESPCR 2000,
89070 Ulm/Donau, Germany. Tel: + 49 (0) 731 50 22251;
Fax: + 49 (0) 731 50 23772; E-mail: ralf.peter@medizin.uni-
ulm.dei; Website: http://www.uni-ulm.de/klinik/dermatologie
10-13 July 2000
29th British Congress of Obstetrics and Gynaecology
International Convention Centre
Birmingham, UK
Further information:
BCOG Secretariat, Congress House, 65 West Drive, Cheam,
Sutton, Surrey SM2 7NB, UK. Tel: + 44 (0) 20 8661 0877;
Fax: + 44 (0) 20 8661 9036; E-mail: info@conforg.com
Erratum
Br J Cancer 81(6): 1088-1093
Reproducibility of microvessel counts in breast cancer specimens
LP Marson, KM Kurian, WR Miller and JM Dixon
Figures 5 and 6 were transposed and Figure 5 should have
appeared as Figure 6, and Figure 6 as Figure 5.
CALENDAR
1246
British Journal of Cancer (2000) 82(6), 1246
(c) 2000 Cancer Research Campaign
Article no. bjoc.1999.1069, available online at http://www.idealibrary.com on

				

